# Fall-Risk-Increasing drugs and falls in community-dwelling older adults: a prospective cohort study in a single Danish municipality

**DOI:** 10.1007/s41999-026-01568-x

**Published:** 2026-08-12

**Authors:** Thorbjørn Irskov Mortensen, Gustav Valentin Gade, Martin Grønbech Jørgensen, Lotta Seppala, Nathalie van der Velde, Tahir Masud, Jesper Ryg

**Affiliations:** 1 Department of Clinical Research, Geriatric Research Unit, University of Southern Denmark, Odense, Denmark; 2 Department of Geriatric Medicine, Odense University Hospital, Odense, Denmark; 3 Department of Geriatric Medicine, Aalborg University Hospital, Aalborg, Denmark; 4 Department of Clinical Medicine, Aalborg University, Aalborg, Denmark; 5 Department of Internal Medicine, Section of Geriatric Medicine, Amsterdam UMC Location University of Amsterdam, Amsterdam, The Netherlands; 6 Amsterdam Public Health Research Institute, Amsterdam, The Netherlands; 7 Nottingham University Hospitals NHS Trust, Nottingham, UK; 8 Department of Internal Medicine Geriatric Section, Geriatric Research and Clinical Evidence (GRACE), Copenhagen University Hospital - Herlev and Gentofte Hospital, Borgmester Ib Juuls Vej 1, Herlev, 2730 Copenhagen, Denmark; 9 Department of Clinical Medicine, University of Copenhagen, Copenhagen, Denmark

**Keywords:** Falls, Recurrent falls, Fall-risk-increasing drugs, Adverse drug events, Older adults, Prospective study

## Abstract

**Purpose:**

Fall-risk-increasing drugs (FRIDs) are a significant and modifiable risk factor for falls in older adults. However, prospective data using robust fall ascertainment on specific FRID classes are scarce in community-dwelling populations. We aimed to investigate the association between individual FRID classes and both single and recurrent falls in older community-dwelling adults.

**Methods:**

We analysed data from a Danish prospective cohort of 241 community-dwelling adults aged ≥ 75 years. Medication records were collected at baseline, and drugs were classified using the STOPPFall consensus FRIDs list. Falls were recorded prospectively for 1 year using monthly fall-calendars, verified using telephone follow-ups. Associations between FRIDs and one-time (1 fall) and recurrent falls (≥ 2 falls) were explored using Cox proportional hazards model, adjusting for clinical and sociodemographic factors.

**Results:**

The median (IQR) age was 82 (80, 86) years, 66.4% were women, and 43.6% had a positive fall history. Prevalences of polypharmacy and use of any FRID were 70.5% and 73.9%, respectively. During follow-up, 88 participants (36.5%) fell at least once while 41 participants (17.0%) fell twice or more. The total number of FRIDs was not associated with falls. Baseline use of benzodiazepine-related drugs, strong anticholinergics, and antiepileptics were significantly associated with recurrent falls in the fully adjusted analyses with hazard ratios (95% confidence intervals) of 3.34 (1.28–8.75), 2.48 (1.04–5.89), and 4.78 (1.24–18.35), respectively.

**Conclusion:**

Several specific FRID classes were significantly associated with recurrent falls in this cohort of well-functioning community-dwelling adults, highlighting the importance of targeted medication review and careful prescribing strategies.

## Key summary points

**Aim:**

To explore the association between specific classes of fall-risk-increasing drugs and both one-time (1 fall) and recurrent (≥ 2) falls in community-dwelling adults aged 75 and older, utilizing prospective fall ascertainment and adjusting for relevant confounders.

**Findings:**

Benzodiazepine-related drugs, strong anticholinergics, and antiepileptics were each significantly associated with recurrent falls when adjusting for clinical and sociodemographic factors in this cohort of well-functioning community-dwelling older adults.

**Message:**

Fall-risk-increasing drugs are an important modifiable risk factor for recurrent falls among older adults, and identifying individuals at risk is essential when conducting medication reviews or prescribing for this patient group.

## Introduction

Falls represent a significant health concern among older adults due to their high prevalence and severe consequences. Approximately one-third of community-dwelling adults aged 65 years and older experience one or more falls annually, which rises to half among those aged 80 and older [1, 2]. Additionally, falls are linked to considerable morbidity and mortality, thereby placing a substantial burden on healthcare systems due to the high cost associated with direct and indirect expenses [3, 4]. This highlights the importance of effective fall prevention strategies.

The etiology of falls is often multifactorial, as numerous risk factors have been identified [5]. Among these, Fall-Risk-Increasing Drugs (FRIDs) remain an important and modifiable risk factor. In 2021, the STOPPFall study identified 14 classes of drugs as FRIDs by international expert consensus, using a Delphi technique [6]. These include medications commonly used by older adults, such as diuretics and anticholinergics [7, 8], highlighting the need for targeted strategies to address the risk associated with FRIDs in this population. In line with this, the recent World Falls Guidelines emphasize the importance of systematically evaluating FRIDs during medication reviews aimed at preventing falls and when prescribing medications for older adults [9].

A recent series of systematic reviews and meta-analyses explored the association between various potential FRID classes and falls [10–12]. However, these reviews identified significant variability in methodological approaches, reflected in the considerable heterogenicity of synthesized results. Notably, a large part of the included studies lacked robust methods for fall ascertainment, as many used long-interval calendars or extended recall periods, increasing the risk of recall bias. Moreover, investigations into associations with recurrent falls were very scarce for several potential FRID classes. Additionally, the majority of included studies did not use transparent medication classifications, leaving many FRID classes insufficiently examined in community-dwelling older adults using rigorous fall ascertainment and consistent medication classification. Lastly, the studies demonstrated inconsistencies in the selection and application of confounders in their multivariable analyses, with some failing to adjust for confounders entirely and others varying substantially in their choices [10–12]. Consequently, an overarching recommendation was that future observational studies should address these methodological limitations in both their design and execution.

Addressing these important gaps through well-controlled prospective studies is essential to improve fall prevention guidelines, and advancing knowledge within this area. Therefore, this prospective cohort study aims to investigate the association between individual FRID classes and both one-time and recurrent falls in community-dwelling adults aged 75 and above while adjusting for appropriate confounders.

## Methods

This was a single center prospective cohort study of older adults living in the community and followed for 12 months. The primary outcome of interest was falls at follow-up with the scope of the paper to assess the association between FRIDs at baseline and one-time falls (1 fall) or recurrent falls (≥ 2 falls). Reporting of the study followed STROBE (Strengthening the Reporting of Observational Studies in Epidemiology) guidelines [13].

### Setting and study population

The setting and participant characteristics have been described in detail elsewhere [14]. In short, community-dwellers aged 75 years or older residing in one Danish municipality (Hjørring, Jutland) were eligible for inclusion from June 2018 to July 2019. Participants were recruited through preventive home visits by community nurses, senior activity centers, and other senior societies and assessed for eligibility during a follow-up preventive home visit or at the recruiting site. Exclusion criteria were self-reported acute illness in the seven days prior to inclusion, inability to stand unassisted for one minute, unable to understand Danish, or having a known dementia diagnosis. Participants were followed until death, moving outside the municipality, or end of study, which ever came first.

### Falls

Falls were defined according to international nomenclature as *an unexpected event in which the participants come to rest on the ground, floor, or lower level* [15] and prospectively recorded by patients returning monthly fall-calendars [16]. Telephone follow-ups were conducted to confirm reported falls, gather details about the events, and document any resulting injuries. Participants were categorized according to prospective falls into non-fallers (0 falls), one-time fallers (1 fall only), or recurrent fallers (≥ 2 falls).

### Fall-risk-increasing drugs

Upon enrollment, participant medication records were obtained using the electronic Shared Medication Record. Use of FRIDs was defined using the STOPPFall tool [6] and recorded as total number of FRIDs as well as the specific FRID classes. In addition, we subdivided the *anticholinergics* category according to the anticholinergic cognitive burden scale (ACB) [17]. Before data analyses, all medications were manually classified as belonging to a FRID class or not using the following predefined Anatomical Therapeutic Chemical (ATC)-codes: Benzodiazepines (BZs), (N03AE, N05BA, N05CD); Antipsychotics (N05A); Benzodiazepine-related drugs (BZRDs) (N05CF); Opioids (N02A, R05DA); Antidepressants (N06A); Anticholinergics, ACB score of 1 (A02BA01, A02BA02, A07DA03, B01AA03, B01AC07, C01AA05, C01DA14, C03CA01, C07AB02, C07AB03, C08CA05, C09AA01, H02AB07, H02AB09, N02AA01, N02AB03, N05AD01, N05AH05, N05AX08, N05AX12, N05AX13, N05BA01, N05BA12, N06AB08, N06AX16, R03DA04, R05DA04, R06AE07, R06AE09, R06AX13, R06AX27); Anticholinergics, ACB score of 2 (N03AF01, N03AF02, N04BB01, N05AG02); Anticholinergics, ACB score of 3 (A03AB05, A03BA01, A03BA03, A03BB01, A04AD01, G04BD02, G04BD04, G04BD07, G04BD08, G04BD09, G04BD10, G04BD11, N04AA, N04AB, N05AA01, N05AB03, N05AC02, N05AH02, N05AH03, N05AH04, N05BB, N06AA, N06AB05, R06AA, R06AD02, R06AE05); Antiepileptics (N03); Diuretics (C03, C07B, C07C, C09BA, C09DA, C09DX01); Alpha-blockers used as antihypertensives (C02CA); Alpha-blockers for prostate hyperplasia (G04CA); Centrally-acting antihypertensives (C02A); Antihistamines (N07CA02, R06); vasodilators used in cardiac disease (C01D); and drugs for urinary frequency and incontinence (G04BD, G04CA53). Some drugs could belong to more FRID classes, e.g. nortriptyline belonging in the FRID group of “antidepressants”, “anticholinergic – any ACB”, and “anticholinergic – ACB3”.

### Baseline data

Data were also collected on physical tests and through self-reported questionnaires. Physical tests included the timed 4-m walking test for gait speed [18, 19], unilateral lower limb strength using the Nintendo Wii Balance Board and FysioMeter software [20], and lower limb reaction speed also using the Nintendo Wii Balance Board and FysioMeter software [21]. Physical tests were administered by trained nurses during preventive home visits, healthcare workers at senior activity centers, and by research personnel and municipality staff at the local community center of Hjørring. Self-reported data included demographics, prior falls within the last year, self-perceived fall risk (“Do you think you will fall within the next year?”), self-reported dizziness (“Do you occasionally experience dizziness?”), and the following standard questionnaires: the short Orientation-Memory-Concentration test (OMC) to assess cognition [22], the Tilburg Frailty Indicator (TFI) and Vulnerable Elders Survey-13 (VES) for frailty and disability [23, 24], the short-form Mini Nutritional Assessment (MNA) for nutritional status [25], the short Falls Efficacy Scale International for concern of falling [26], the Geriatric Depression Scale-4 (GDS) for depressive symptoms [27], and the EuroQol Five Dimensions Three Levels (EQ-5D-3L) for quality of life. Comorbidity was assessed using the Charlson Comorbidity Index [28], derived from ICD-10 diagnoses recorded at baseline.

### Statistics

A sample size calculation was not conducted prior to study initiation; instead, recruitment feasibility during the inclusion period determined the sample size, and post hoc power analysis were performed to estimate the sample size required to achieve 80% power based on the observed effect sizes. Variables were assessed for normality through visual inspection of histograms and quantile–quantile plots. If normality appeared plausible but not clearly established, the Shapiro–Wilk test was applied. The proportions of one-time fallers and recurrent fallers were calculated, along with the proportion of participants using medications from each FRID group**.** Descriptive statistics for continuous, non-parametric data were reported using median and interquartile range.

Non-fallers, one-time fallers, and recurrent fallers were compared using non-parametric tests. For variables with an anticipated ordering, a trend test was employed. For variables without expected trends, the Kruskal–Wallis test and Fisher’s T-test were applied. Associations between FRIDs and falls were assessed using Cox proportional-hazards models. Schoenfeld residuals was used to evaluate the proportional-hazards assumption. Calculations were completed for FRID classes with more than five participants. Both univariable and multivariable analyses were conducted to calculate crude and adjusted hazard ratios (HRs) with corresponding confidence intervals (CIs). The following variables were included as covariables in the multivariable analysis due to their established association with fall risk: age [5], sex [5], alcohol consumption [9], education level [29], depressive symptoms [5], self-reported dizziness [5], gait speed ≤ 0.8 m/s [19], prior falls [5, 14], and self-perceived fall-risk [5, 14]. Finally, sensitivity analysis was performed to assess the potential of dizziness and gait speed as effect modifiers as well as taking comorbidity into account and stratifying data for prior falls.

Missing data entries averaged < 1% for the covariables and therefore did not warrant complex imputation. Instead, missing data were managed by exclusion during analysis maintaining statistical power and sample integrity. All data analyses were performed using STATA version 18.5 (StataCorp, College Station, TX, USA). P values < 0.05 were considered statistically significant.

### Ethics

The study was approved by the Danish Data Protection Agency (2018–82) and registered on clinicaltrials.gov (NCT03608709) before initiation. The study was conducted in full compliance with the Helsinki Declaration and Danish law. According to Danish legislation, no permission was required from the ethical committee since the study did not include an intervention. All participants provided explicit oral and written consent before study inclusion.

## Results

The final study population included 241 participants, median (IQR) age of 82 (80, 86) years, mostly women (66.4%). Follow-up ranged from 19 days to 1 year, with a median follow-up time of 365 days, corresponding to a total of 219 person years. Prior falls were seen among 43.6%, 22.4% had alcohol consumption ≥ 7 units weekly, 51.0% reported dizziness, and 18.8% had self-perceived 1 year fall-risk. Participants were relatively well-functioning exhibiting low scores on TFI and VES-13 along with a high OMC score. Furthermore, 94.6% had GDS < 2 points and 65.6% a gait speed > 0.8 m/s. Prevalence of polypharmacy and use of any FRID was 70.5% and 73.9%, respectively with a median (IQR) number of prescribed any medications of 7 (4, 10) and any FRIDs of 1 (0, 2). Baseline characteristics of the cohort are summarized in Table 1. Additional baseline data and the participant flow diagram have previously been published [14].

**Table 1:** Baseline characteristics for the overall cohort and stratified by outcome groups (non-fallers (0 falls), one-time fallers (1 fall only), or recurrent fallers (≥ 2 falls)), with group comparison analyses

Variable and summary measure	Total (*n* = 241)	Non-faller (*n* = 153)	One-time faller (*n* = 47)	Recurrent faller (*n* = 41)	P-value
*Demographics*					
Age (years), median [IQR]	82 [80, 86]	82 [80, 86]	84 [80, 87]	81 [76, 85]	0.850
Women, *n* (%)	160 (66.4)	101 (66.0)	33 (70.2)	26 (63.4)	0.817
Alcohol consumption ≥ 7 units weekly, *n* (%)	54 (22.4)	28 (18.3)	13 (27.7)	13 (31.7)	0.039 *
Level of education, *n* (%)					
Municipal primary or lower secondary school	106 (44.0)	75 (49.0)	17 (36.2)	14 (34.2)	
Skilled worker	68 (28.2)	43 (28.10)	16 (34.0)	9 (22.0)	
General upper secondary or higher	67 (27.8)	35 (22.9)	14 (29.8)	18 (43.9)	0.010 *
Experience dizziness, *n* (%)	123 (51.0)	69 (45.1)	25 (53.2)	29 (70.7)	0.004 **
Fall within last year, *n* (%)	105 (43.6)	53 (34.6)	22 (46.8)	30 (73.2)	< 0.001 ***
Self-perceived fall-risk ^a^, *n* (%)	45 (18.8)	19 (12.5)	6 (13.0)	20 (48.8)	< 0.001 ***
*Medication & comorbidity*					
Drugs, median [IQR]	7 [4, 10]	6 [4, 10]	6 [4, 9]	7 [5, 12]	0.185
FRIDs, median [IQR]	1 [0, 2]	1 [1, 2]	1 [0, 2]	1 [0, 3]	0.852
Polypharmacy (≥ 5 prescribed drugs), *n* (%)	170 (70.5)	105 (68.6)	32 (68.1)	33 (80.5)	0.263
Charlson comorbidity index, score grouping, *n* (%)					
0	106 (44.0)	69 (45.1)	24 (51.1)	13 (31.7)	
1–2	108 (44.8)	67 (43.8)	17 (36.2)	24 (58.5)	
≥ 3	27 (11.2)	17 (11.1)	6 (12.8)	4 (9.8)	0.483
*Test scores*					
Orientation-memory-concentration test (best score; 28), median [IQR]	26 [22, 26]	26 [22, 28]	26 [20, 26]	25 [24, 26]	0.772
Short-form mini-nutritional assessment (best score; 14), median [IQR]	13 [12, 14]	13 [12, 14]	13 [12, 14]	13 [11, 14]	0.332
Short falls efficacy scale-International (best score; 7), median [IQR]	8 [7, 11]	8 [7, 11]	8 [7, 10]	9 [7, 12]	0.295
Tilburg frailty indicator (best score; 0), median [IQR]	3 [1, 5]	3 [1, 4]	2 [2, 4]	3 [2, 7]	0.026 *
Vulnerable elders survey 13 (best score; 0), median [IQR]	3 [1, 4]	2.5 [1, 4]	3 [1, 3]	3 [1, 6.5]	0.361
Geriatric depression Scale – 4 item ≥ 2 points, *n* (%)	13 (5.4)	8 (5.2)	0 (0.0)	5 (12.2)	0.036*
EQ-5D-3L (range −0,167–1.0), median [IQR]	0.7 [0.6, 1.0]	0.7 [0.6, 1.0]	0.7 [0.6, 1.0]	0.7 [0.6, 1.0]	0.286
*Physical test scores*					
Gait speed ≤ 0.8 m/s, *n* (%)	83 (34.4)	53 (34.6)	16 (34.0)	14 (34.2)	0.940
Total isometric lower limb strength (kg), median [IQR]	132.8 [89.2, 189.8]	134.8 [91.6, 192.5]	121 [88.1, 170.0]	147.7 [94.5, 194.3]	0.639
Average lower limb reaction speed (ms), median [IQR]	1315 [1122, 1567]	1322 [1126, 1605]	1256 [1106, 1516]	1334 [1134, 1566]	0.468

*FRIDs* fall-risk-increasing drugs, *IQR* interquartile range

*p < 0.05, ** *p* < 0.01, *** *p* < 0.001

^a^ Do you think you will fall again within 365 days?

**Fig. 1:**
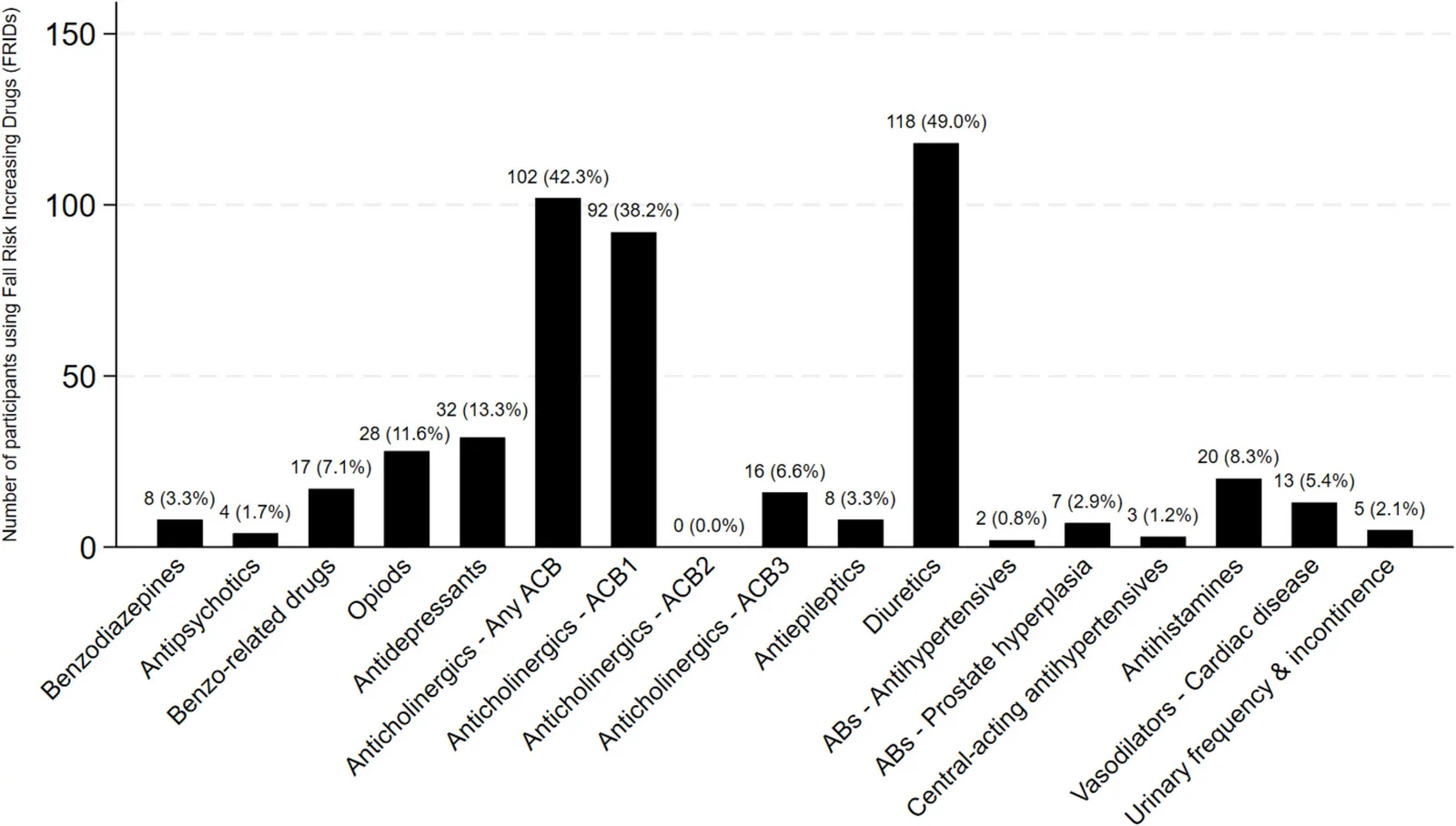
Proportion of participants using medications from each FRID class. *ACB* anticholinergic cognitive burden scale, *Benzo-related drugs*: benzodiazepine-related drugs

The most prevalent FRID classes were diuretics (49.0%) and anticholinergics with any ACB score (42.3%). Proportions of participants using medication across all FRID classes are illustrated in Fig. 1. Participants not completing 1 year follow-up (*n* = 43) were significantly older (median (IQR) 85 (81, 87) years vs 82 (79, 86) years, *p* = 0.019), had higher Charlson comorbidity score (median (IQR) 1 (0, 2) vs 1 (0, 2), *p* = 0.035), and used more medications (median (IQR) 8 (6, 12) vs 6 (4, 9), *p* < 0.001) compared to those with 1 year follow-up.

### FRIDs and falls

During follow-up, 88 participants (36.5%) fell at least once while 41 participants (17.0%) fell twice or more. The fall rate was 0.94 falls per person-year. Non-fallers, one-time fallers, and recurrent fallers differed significantly in several characteristics: alcohol consumption (*p* = 0.039), level of education (*p* = 0.010), dizziness (*p* = 0.004), previous falls (*p* < 0.001), self-perceived fall-risk (*p* < 0.001), depressive symptoms as measured by GDS (*p* = 0.036), and frailty measured by TFI (*p* = 0.026) (Table 1).

Crude and adjusted HRs and CIs for one-time falls and recurrent falls are presented in Table 2 for all FRID classes comprising medications used by more than five people. The total number of FRIDs was not significantly associated with one-time falls or recurrent falls in neither the unadjusted nor the adjusted model. In addition, no individual FRID classes were significantly associated with one-time falls in neither of the models. However, benzodiazepine-related drugs (HR 2.49, 95% CI 1.05–5.92) and anticholinergics with an ACB score of 3 (HR 2.92, 95% CI 1.29–6.59) were both significantly associated with recurrent falls in the univariable model. Furthermore, benzodiazepine-related drugs, anticholinergics with an ACB score of 3, and antiepileptics remained significantly associated with the risk of recurrent falls in the fully adjusted model (HR 3.34, 95% CI 1.28–8.75; HR 2.48, 95% CI 1.04–5.89; and HR 4.78, 95% CI 1.24–18.35, respectively).

**Table 2:** Estimated hazard rates, 95% confidence intervals, and post hoc statistical power for falls and recurrent falls among the specific fall-risk-increasing drug classes with > 5 participants. Power estimates are reported for analyses with no statistically significant association

One-time faller (1 fall)			
Variable	Univariable model HR (95% CI)	Multivariable model HR (95% CI)	Power %
Total FRIDs	0.92 (0.74–1.15)	0.85 (0.66–1.09)	−
Benzodiazepines ^a^	NA	NA	NA
Benzodiazepine-related drugs	0.73 (0.18–2.99)	0.60 (0.14–2.55)	5.95
Antipsychotics ^a^	NA	NA	NA
Opioids	0.38 (0.09–1.57)	0.34 (0.08–1.44)	28.95
Antidepressants	0.89 (0.35–2.26)	0.68 (0.25–1.81)	6.16
Anticholinergics – Any	1.25 (0.70–2.23)	1.12 (0.60–2.10)	5.19
Anticholinergics – ACB1	1.36 (0.76–2.43)	1.25 (0.66–2.37)	16.27
Anticholinergics – ACB2 ^a^	NA	NA	NA
Anticholinergics – ACB3	0.43 (0.06–3.14)	0.35 (0.05–2.58)	14.36
Antiepileptics ^a^	1.76 (0.45–7.25)	1.67 (0.37–7.64)	13.94
Diuretics	0.79 (0.44–1.40)	0.70 (0.38–1.30)	14.78
Alpha-blockers used as antihypertensives ^a^	NA	NA	NA
Alpha-blockers used for prostate hyperplasia ^a^	NA	NA	NA
Centrally-acting hypertensives ^a^	NA	NA	NA
Antihistamines	0.99 (0.36–2.76)	0.88 (0.31–2.49)	2.59
Vasodilators used for cardiac disease ^a^	0.78 (0.17–8.76)	0.75 (0.18–3.12)	3.98
Drugs for urinary infrequency and incontinence ^a^	NA	NA	NA

Recurrent faller (≥ 2 falls)			
Variable	Univariable model HR (95% CI)	Multivariable model HR (95% CI)	Power %
Total FRIDs	1.12 (0.92–1.37)	0.99 (0.78–1.24)	−
Benzodiazepines ^a^	0.62 (0.09–4.52)	0.45 (0.05–3.66)	3.98
Benzodiazepine-related drugs	2.49 (1.05–5.92) *	3.34 (1.28–8.75) *	−
Antipsychotics ^a^	NA	NA	NA
Opioids	1.84 (0.88–3.85)	0.91 (0.40–2.06)	44.17
Antidepressants	1.51 (0.67–3.40)	0.96 (0.39–2.38)	9.37
Anticholinergics – Any	1.70 (0.92–3.15)	1.38 (0.68–2.81)	41.6
Anticholinergics – ACB1	1.56 (0.84–2.88)	1.30 (0.63–2.67)	28.17
Anticholinergics – ACB2 ^a^	NA	NA	NA
Anticholinergics – ACB3	2.92 (1.29–6.59) *	2.48 (1.04–5.89) *	−
Antiepileptics ^a^	3.08 (0.95–10.00)	4.78 (1.24–18.35) *	−
Diuretics	0.70 (0.38–1.31)	0.70 (0.35–1.41)	23.16
Alpha-blockers used as antihypertensives ^a^	NA	NA	NA
Alpha-blockers used for prostate hyperplasia ^a^	2.50 (0.77–8.09)	3.64 (0.95–13.88)	30.01
Centrally-acting hypertensives ^a^	NA	NA	NA
Antihistamines	1.05 (0.37–2.95)	0.74 (0.24–2.27)	5.52
Vasodilators used for cardiac disease ^a^	1.44 (0.44–4.67)	1.32 (0.37–4.69)	7.18
Drugs for urinary infrequency and incontinence ^a^	NA	NA	NA

Multivariable analysis adjusted for age (years), sex (male/female), alcohol consumption (≥ 7 units per week), education level (municipal primary and lower secondary school or skilled worker or general upper secondary and longer education), depressive symptoms (GDS score of ≥ 2), self-reported dizziness (yes/no), gait speed ≤ 0.8 m/s (yes/no), prior falls (≥ 1 falls within last year), and self-perceived fall risk (yes/no)

*ACB* anticholinergic cognitive burden scale, *FRIDs* fall-risk-increasing drugs

* *p* < 0.05, ** *p* < 0.01, *** *p* < 0.001

^a^ denotes ≤ 15 participants in the FRID group

**Fig. 2:**
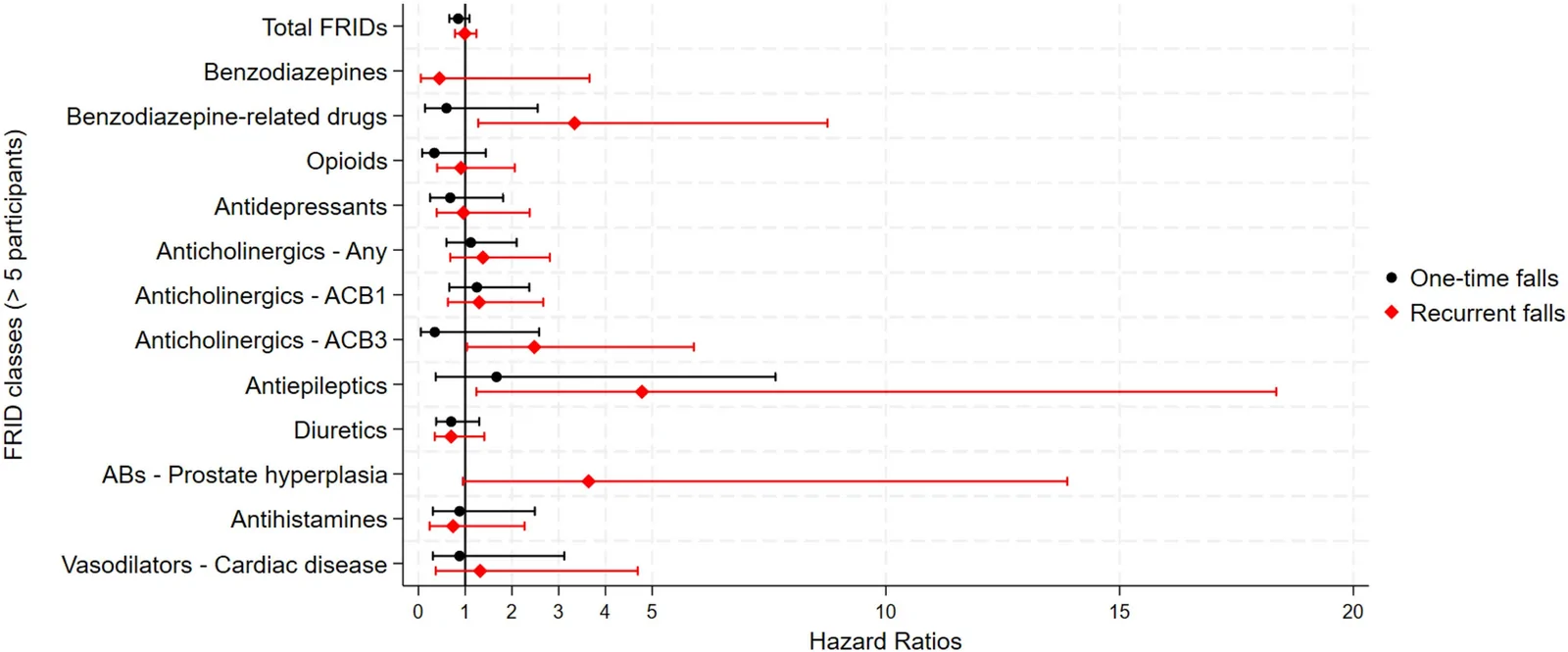
Forest-style plot depicting the fully adjusted estimated hazard ratios and corresponding confidence intervals for falls and recurrent falls, for all FRID classes with > 5 participants. Lines (95% confidence intervals) with round dots (hazard rates) represent one-time falls (1 fall) and lines with squares represent recurrent falls (≥ 2 falls). Estimated hazard rates and confidence intervals were unable to be calculated in the FRID classes benzodiazepines and alpha-blockers used for prostate hyperplasia, for one-time falls. The model adjusted for age (years), sex (male/female), alcohol consumption (≥ 7 units per week), education level (municipal primary and lower secondary school or skilled worker or general upper secondary and longer education), depressive symptoms (GDS score of ≥ 2), self-reported dizziness (yes/no), gait speed ≤ 0.8 m/s (yes/no), prior falls (≥ 1 falls within last year), and self-perceived fall risk (yes/no). ABs: alpha-blockers, ACB: anticholinergic cognitive burden scale, FRID: fall-risk-increasing drugs

The fully adjusted HRs and CIs for one-time falls and recurrent falls across FRID classes are shown in Fig. 2.

### Sensitivity and post hoc analyses

The overall associations between FRIDs and falls as described in the prior section did not change when performing sensitivity analyses by excluding potential mediators of dizziness and gait speed from the models. Accounting for potential confounding by indication through comorbidity adjustment or stratification by prior falls did also not alter the results.

Post hoc power analysis suggested that the study was underpowered for the individual FRID class analyses, limiting the ability to detect smaller effect sizes. Furthermore, post hoc sample size calculations based on the observed effect sizes for total FRID use indicated that approximately 2755 participants for one-time fallers and 3589 participants for recurrent fallers would have been required. These estimates highlight the limited sample size available for the present analyses and should be considered when interpreting the findings. The results of the post hoc power analyses are presented in Table 2 for all FRID classes with no significant association with one-time or recurrent falls.

## Discussion

In this prospective cohort study of relatively well-functioning community-dwelling older adults, the total number of FRIDs was not significantly associated with one-time falls or recurrent falls. Furthermore, no FRID class was associated with one-time falls. Importantly, we found that several FRID classes were significantly associated with recurrent falls even after taking important confounders into account.

FRIDs are a well-established risk factor for falls in older adults and encompass several drug classes commonly used in this population [6–8, 10–12]. However, only a few previous studies have examined the total number of FRIDs and their association with falls. A recent analysis of a large multinational European study used STOPPFall to define FRIDs and found that the use of at least one FRID, and particularly the use of multiple FRIDs (≥ 2), was linked to higher rates of total-, injurious-, and recurrent falls over 3 years, after adjusting for confounders [30]. Similarly, recently published results from The Irish Longitudinal Study on Ageing (TILDA) prospective cohort also using STOPPFall to define FRIDs, reported an association between use of multiple FRIDs (≥ 2) and both falls and injurious falls when adjusting for confounders, with a median follow-up time of 8 years (range 2–12 years). Subgroup analysis on a four-year follow-up period demonstrated similar findings [31]. Lastly, a large retrospective Swedish case–control study, which included 64,999 cases and 257,596 controls matched by sex, age, and location, reported increasing odds of injurious falls with increasing use of FRIDs [32]. In contrast, we found no association between the total number of FRIDs and the risk of neither one-time nor recurrent falls in our study. This discrepancy may, in part, be attributable to differences in fall ascertainment methods, populations, study design, length of follow-up, and statistical power. Furthermore, the Irish and Swedish studies used retrospective fall ascertainment, whereby falls and injurious falls were recorded at two-year intervals using participant recall or hospital discharge diagnoses based on ICD-10 [31, 32], contrasting the prospective self-reported data validated by telephone used in our study. In addition, the case–control study was also conducted prior to the development of the STOPPFall tool and reported unadjusted odds [32], whereas our study adjusted for several important factors. Thus, potential confounding and variation in FRID classification may also contribute to the divergent findings. Finally, the two most recent studies included relatively well-functioning populations with baseline characteristics comparable to ours; however, one study assessed falls after 3 years and the other at a median of 8 years, limiting comparability to our results with 1-year of follow-up [30, 31].

In our study, several FRID classes were associated with significant risk of recurrent falls. The association between antiepileptics and recurrent falls across our adjusted models is in line with existing literature among community-dwelling adults [33–35]. While studies examining the association between risk of falls or recurrent falls and anticholinergic drugs as a group have shown inconsistent results [36–38], studies examining the cumulative anticholinergic burden indicate a positive association between higher anticholinergic burden and the risk of falls [39–41]. These findings align with our results showing an increased risk of recurrent falls for participants receiving anticholinergic drugs with a high ACB score. Interestingly, we found that the use of BZRD was associated with recurrent falls, while BZs themselves were not. These findings differ from those of a recent systematic review and meta-analysis, which showed an association between BZs and falls, while BZRD were generally not associated with falls in most studies [11]. This might partly be explained by differences in geographical and healthcare-related settings, the type of fall assessed, and variations in the inclusion or adjustment for covariables. Perhaps even more importantly, Danish healthcare policies targeting the use of potentially addictive drugs have led to a marked decline in BZ use and a shift toward BZRDs [42]. This shift could imply more careful and selective BZ prescribing, while BZRDs might have become the more commonly used hypnotic, also including in older populations. Although our data does not allow us to explore this further, it underscores that while limiting BZ use is important, BZRDs also carry safety risks [43], highlighting the need for research into safer alternatives.

Similarly, the divergence between our findings and the international literature regarding BZs and BZRDs highlights the broader methodological challenges of applying internationally derived frameworks such as STOPPFall to regional cohorts. Prescribing practices are influenced by national guidelines, healthcare policies, and local clinical traditions [44], which may affect the distribution and selection of specific FRID classes within a given population. Consequently, when international frameworks are applied to national cohorts, differences in prescribing patterns may influence the observed associations. While STOPPFall has been incorporated into Danish deprescribing and fall prevention guidelines [45, 46] and broadly reflects medication classes used in Denmark, prescribing decisions remain influenced by factors that were not captured in our dataset [44]. These factors may partly explain why some established FRID classes were not associated with falls in the present study. However, such considerations do not diminish the value of standardized international frameworks such as STOPPFall, which remain important for comparability across studies and settings. Instead, they underline the importance of interpreting findings within their local prescribing context and highlight the need for future studies examining how national prescribing patterns and healthcare policies may modify associations between FRIDs and falls.

Our study population was relatively well-functioning for their age group, with test scores aligning with typical healthy values and indicating low fall risk [22, 23, 25–27]. This is reflected in the observed fall rate of 36.5%, which is notably lower than the expected 50% for aged-matched community-dwelling adults like our cohort with a median age of 82 years [1, 2]. This might also partly explain our findings of no association between FRIDs classes and one-time falls, or between total number of FRIDs and one-time falls or recurrent falls. However, our findings suggest that recurrent fallers may represent a distinct, high-risk subgroup more susceptible to the fall-risk-increasing effects of FRIDs, potentially due to differences in underlying risk factors or vulnerabilities. This finding aligns well with the current World Fall Guidelines, which classify individuals with recurrent falls (≥ 2 in 12 months) as high-risk and recommend multifactorial assessments, including medication reviews to deprescribe FRIDs [9]. The potential distinction between one-time fallers and recurrent fallers may also explain the absence of significant associations between FRID classes and one-time falls, underscoring the need for tailored fall prevention strategies. Furthermore, our study underscores the importance of caution when prescribing or reviewing medications such as BZRDs, anticholinergics with a high ACB score, and antiepileptic drugs for older community-dwelling adults with a history of recurrent falls, as previously suggested [6]. Specifically, potent anticholinergics with an ACB score of 3 were associated with a significantly increased risk of recurrent falls, independent of the cumulative anticholinergic burden. These findings may inform deprescribing guidelines aimed at preventing recurrent falls in older adults. In support of this, further studies should be undertaken to investigate individual ATC-codes within these categories, which will improve future guidelines further and assist clinicians in making tailored prescribing choices within shared decision-making settings [47].

Our study had several strengths. First, we used recommended definitions of falls and fall ascertainment methods increasing the external validity. Furthermore, we used prospective falls data with short interval reporting and ensured verification of the reported falls reducing the risk of recall bias [15, 16]. Second, we adopted evidence-based medication classifications in accordance with STOPPFall as recommended by the recent World Fall Guideline [6, 9], and provided ATC-codes for each FRID group to ensure transparency. Third, extensive baseline data enabled robust comparisons of outcome groups and allowed us to adjust our multivariable models accordingly. Finally, our dataset contained minimal missing data, and the application of survival time analysis allowed us to handle loss-to-follow-up appropriately.

However, our study also had some important limitations. First, half of the study population was not consecutively sampled due to low recruitment rate, resulting in risk of selection bias that may influence the internal validity of the population. Furthermore, the limited sample size resulting from the low recruitment rate prevented inclusion of all FRID classes in the class-specific analyses and may have reduced the precision of estimates for those that were included. Also, post hoc sample size calculations based on the observed effect sizes for total FRID use indicated that substantially larger sample sizes would have been required for the analyses of both one-time and recurrent falls. These estimates suggest an increased risk of type II error, limited precision of the estimates, and reduced generalizability of the findings. Second, confounders were measured only at baseline, meaning significant changes in covariable predictors during the study period may not have been captured. Similarly, since medication data was collected solely at baseline, changes in FRID usage, such as discontinuation, initiation, or modification of treatment, may not have been reflected. However, both aspects are reflecting the nature of cohort studies. Also, while structured clinical follow-up visits at fixed intervals may have provided additional opportunities for exposure and outcome assessments, the monthly fall calendar combined with telephone follow-up represented a validated and resource-efficient approach to fall ascertainment. However, although baseline cognitive screening suggested no meaningful impairment, the absence of a comprehensive cognitive evaluation means that undetected cognitive impairment cannot be fully excluded. Third, while our dataset holds information on regularly taken FRIDs, no information was available on dosage or duration of use, preventing any investigations of potential dose–response relationships. Fourth, the sample size was insufficient to isolate users of individual FRID classes, possibly allowing fall-risk increasing properties from other FRIDs to influence the results in FRID subgroup analysis. Due to limited statistical power, adjusting for concurrent FRID use of other FRIDs within the category-specific analysis was not feasible, as doing so risked model overfitting. However, the median number of FRIDs was low and did not differ significantly between groups, suggesting the overlapping effects may have a limited impact on the study findings. Additionally, certain FRID classes had no or very few participants, limiting the external validity of the estimated associations. Finally, individuals who experience adverse effects may discontinue treatment, resulting in a selective retention of those who tolerate the medication well. As the indication for FRID use was unavailable in our dataset, residual confounding by indication or contraindication remains possible. Nevertheless, adjustment for comorbidity did not alter our findings. Also, while adjusting for comorbidity using Charlson Comorbidity Index may not fully capture the fall risk attributable to specific underlying conditions, potentially resulting in residual confounding, several covariables included in the multivariable model reflect clinical manifestations and functional consequences of such conditions that are independently associated with fall risk. These variables may therefore partially account for the influence of underlying disease burden, although residual confounding cannot be excluded. Collectively, these limitations suggest that the findings should be interpreted with caution and that their generalizability beyond similar populations may be limited.

## Conclusion

Our findings indicate that several FRIDs are associated with prospective recurrent falls, even in a population of relatively well-functioning individuals with low FRID use, after adjusting for key confounders. However, some well-established FRID classes were not linked to falls in our study, underscoring the complexity of medication-related fall risk. These results highlight the importance of identifying individuals at risk when conducting medication reviews and a need for future research into specific ATC-codes to better inform future fall prevention guidelines.

## Data and materials availability

Data used in the analysis for the present study are not available outside Denmark due to national legislation. Inside the country, the corresponding author can be contacted.

## Data Availability

Not applicable.
